# Neurophysiological Characteristics of Allgrove (Triple A) Syndrome: Case Report and Literature Review

**DOI:** 10.1177/2329048X211031059

**Published:** 2021-10-04

**Authors:** Daniel I. Weiman, Meredith K. Gillespie, Taila Hartley, Matthew Osmond, Yoko Ito, Care4Rare Canada Consortium, Kym M. Boycott, Kristin D. Kernohan, Matthew Lines, Hugh J. McMillan

**Affiliations:** 1 12365 University of Ottawa, Ottawa, ON, Canada; 2 Children’s Hospital of Eastern Ontario Research Institute, 274065 University of Ottawa, Ottawa, ON, Canada; 3 Newborn Screening Ontario, Ottawa, ON, Canada; 4 Alberta Children’s Hospital, Calgary, AB, Canada

**Keywords:** polyneuropathies pyramidal tracts, achalasia, addisonianism alacrima syndrome, muscle weakness, autonomic nervous system electromyography

## Abstract

Allgrove or “Triple A” syndrome is characterized by alacrima, achalasia, and adrenocorticotropic hormone-resistant adrenal insufficiency, as well as central and peripheral nervous system involvement. Patients demonstrate heterogeneity with regard to their age of symptom onset, disease severity, and nature of clinical symptoms. Neurophysiological testing has also shown variability ranging from: motor neuron disease with prominent bulbar involvement, motor-predominant neuropathy, or sensorimotor polyneuropathy with axonal or mixed axonal and demyelinating features. We report an 11-year-old boy who presented with neurological symptoms of progressive spasticity and peripheral neuropathy. His neurophysiological testing confirmed a sensorimotor polyneuropathy with axonal and demyelinating features. Exome sequencing identified compound heterozygote variants in the *AAAS* gene. We summarize the neurophysiological findings in him and 29 other patients with Allgrove syndrome where nerve conduction study findings were available thereby providing a review of the heterogeneity in neurophysiological findings that have been reported in this rare disorder.

## Introduction

Allgrove syndrome is characterized by the triad of alacrima, achalasia, and adrenocorticotropic hormone (ACTH) resistant adrenal insufficiency (Addison disease), denoted by the moniker “triple A syndrome.” Neurological symptoms are common in adolescents and adults which can bring patients to the attention of a neurologist for one or more of: peripheral neuropathy, cognitive decline, progressive spasticity, dysautonomia and/or cranial neuropathies.^1-3^ This multisystem disease results from biallelic pathogenic variants in the *AAAS* (also known as *ALADIN*) gene which belongs to a family of WD repeat-containing proteins localizing to the nuclear envelope.^4^ These nuclear pore complexes facilitate nucleocytoplasmic transport and regulate cell cycle progression and gene expression.^5^

Allgrove syndrome patients vary with regard to their clinical severity, manifestations, and age of onset, even among individuals sharing the same causative DNA variant.^6^ Phenotypic variability has also been observed among patients with peripheral nerve involvement. Patients have presented in early adulthood with progressive bulbospinal amyotrophy^7^ and concomitant upper and lower motor neuron findings that mimics amyotrophic lateral sclerosis.^8,9^ Neurophysiological testing has also confirmed a phenotype consisting purely of motor nerve involvement in some patients,^10^ while others have been reported to have a sensorimotor polyneuropathy with purely axonal^3^ or mixed axonal and demyelinating features.^1^

We present an 11-year-old boy who was referred with spastic gait and distal weakness. Neurophysiological testing demonstrated a sensorimotor polyneuropathy with axonal and demyelinating features. His findings serve to emphasize the broad spectrum of neurophysiological findings that can be associated with Allgrove syndrome, as well as the importance of maintaining a high index of suspicion for the nonneurological features of this disease.

## Case

An 11-year-old boy presented with slowly progressive distal weakness and abnormal gait noted at 6 to 7 years of age. He was born at term after an uneventful pregnancy to nonconsanguineous parents. His early neurodevelopment was normal. His weakness had become apparent in distal muscles as he had increased difficulty tying shoelaces and printing letters. He also reported generalized fatigue that did not show any diurnal fluctuation and made it difficult for him to keep up with his peers during physical activity. He developed bilateral, nonfluctuant ptosis but no diplopia, dysphasia, or dysarthria. He had no pain or sensory symptoms. Learning difficulties were first noted at age 7 years old, and he was subsequently held back 2 grades in school. When assessed at 11 years of age, he was in a grade 6 classroom but functioning at a grade 1 to 2 level. Medical history was significant for hospitalizations for minor respiratory infections from about 6 to 7 years of age. He had strabismus which was surgically corrected at 11 years old.

Physical examination revealed a nondysmorphic male with conjunctival erythema and irritation from alacrima. His growth was reassuring with head circumference at the 85%ile. Cranial nerve testing was significant for bilateral ptosis and weakness of his orbicularis oculi and oris. Muscle power testing (Medical Research Council grading) revealed intact neck extensor strength but weakness to: neck flexors (4/5), deltoid (4/5), flexor pollicis longus (4-/5), abductor pollicis brevis (4/5), first dorsal interossei (4-/5) abductor digiti minimi (3/5). Although proximal leg strength was preserved, he showed weakness to: tibialis anterior, gastrocnemius, tibialis posterior, peroneus longus, and toe flexors (all 4/5); extensor hallicus longus (2/5). His deep tendon reflexes were brisk at: biceps and triceps (3 + ), brachioradialis (4 + with spread to finger flexors), patella (3 + ) with ankle jerks still elicited (1 + ) despite weakness. Plantar responses were extensor. Spastic catch was noted at both knees with heel cord tightness evident and early hammer-toeing was apparent. He had a spastic and foot-drop gait. Sensory examination noted decreased sharp sensation below his ankles and decreased vibration sense at the ankles. General examination noted hyperpigmentation in his mouth including the palate, gum, and tongue. He had no hepatosplenomegaly or scoliosis.

Nerve conduction studies demonstrated a sensorimotor polyneuropathy with demyelinating and axonal features (Table 1). Sensory responses were absent to lower extremities and showed decreased amplitude to the upper extremities. Motor responses showed decreased compound motor action potential (CMAP) amplitudes to the right ulnar, tibial, and peroneal nerves prolonged latencies to the upper extremities and slowed conduction velocities to the right peroneal nerve (52.5% of lower limit of normal; LLN) and ulnar nerve (74% of LLN). Electromyography of his right tibialis anterior, vastus lateralis, and first dorsal interosseous showed fibrillation potentials (1 + to 2 + ) and positive sharp waves (2 + ). Recruitment of these muscles revealed large amplitude, polyphasic motor unit action potentials that were firing rapidly. MRI of the brain was unrevealing.

**Table 1.** Neurophysiological Findings at 11 Years of Age.

	Right	Normal^a^
MOTOR:		
Median nerve		
DML (ms) wrist (to APB)	**5.0**	< 4.9
CMAP (mV) wrist/elbow	**4.4/4.3**	> 5.7
CV (m/s)	**43**	> 51
Ulnar nerve		
DML (ms); wrist (to ADM)	**4.8**	< 3.1
CMAP (mV) wrist/b-elbow/a-elbow	**0.6/0.6/0.7**	> 7.0
CV (m/s)	**37**	> 54
Tibial nerve		
DML (ms; ankle-AH)	4.8	< 5.6
CMAP (mV) ankle/knee	**1.8/1.3**	> 6.2
CV (m/s)	**40**	> 45
Peroneal nerve		
DML (ms; ankle-EDB)	4.1	< 5.6
CMAP (mV)	**0.2/0.6/0.5**	> 2.6
CV (m/s)	**21**	> 45
SENSORY:
Median nerve		
PL (ms; wrist-digitII)	**3.8**	< 3.4
SNAP (μV)	**11.1**	> 28
CV (m/s)	**41**	> 58
Ulnar nerve		
PL (ms; wrist-digitV)	**3.4**	< 3.1
SNAP (μV)	**4.5**	> 24
CV (m/s)	**38**	>57
Sural nerve		
PL (ms; calf-latmall)		< 4.2
SNAP (μV)	**NR**	> 9
CV (m/s)		< 43
Peroneal nerve		
PL (ms; anterior		< 4.0
SNAP	**NR**	> 5
CV (m/s)		< 40

**Bold values** are abnormal. All sensory responses are antidromic.

Abbreviations: NR, no response; DML, distal onset motor latency; CMAP, compound motor action potential; CV, conduction velocity; PL, peak onset latency; APB, abductor pollicus brevis; ADM, abductor digiti minimi; AH, abductor hallicus; EDB, extensor digitorum brevis; b-elbow, below elbow; a-elbow, above elbow.

^a^ Normal values from Ryan et al. 2019^11^ except peroneal nerve sensory normal values obtained from our laboratory.

Laboratory investigations revealed an elevated serum creatine kinase of 516 U/L (reference: 27-160); TSH of 7.77 mIU/L (reference 0.74-6.23), with a normal free T4. His morning (8AM) cortisol level was below detectable limits at <7 nmol/L (normal 185-624 nmol/L). Subsequent ACTH stimulation testing confirmed primary adrenal insufficiency. Very long chain fatty acid (VLCFA) was mildly elevated with normal serum phytanic acid levels. Repeat fasting VLCFA was normal and a peroxisomal disorder gene panel with copy number testing (Prevention Genetics, WI) was unrevealing. Microarray and a comprehensive Charcot-Marie-Tooth gene panel (London, ON) was negative.

The patient and his parents were enrolled in the Care4Rare Canada Consortium. Research Ethics Board approval was obtained (CTO REB #1577) and his parents provided free and informed consent to participate in this study. Trio exome sequencing was performed and analyzed by the Care4Rare-SOLVE Canada research program as per previously published methodology^12^ revealing biallelic variants in the *AAAS* gene: NM_01173466.1: c.1041_1063del (p.Leu348ThrfsTer4) and; c.57_58del (p.Tyr19Ter) which were inherited in-trans. Both variants identified are loss of function variants which is a known disease mechanism for this condition;^4^ c.1041_1063del had never been reported in presumed healthy controls, and c.57_58del variant has been seen at a low allele frequency of 0.008% (gnomAD). While the c.1041_1063del variant is novel, the c.57_58 variant has been previously identified in trans with other known pathogenic variants in *AAAS* in 2 affected individuals in the literature.^13,14^ Both variants were confirmed in a clinical diagnostic laboratory and formally classify as pathogenic by ACMG criteria and based on the clinical and molecular evidence we conclude that this patient has Allgrove syndrome.

## Discussion

Neurophysiological testing can demonstrate considerable phenotypic variability among patients with genetically-confirmed Allgrove syndrome. This can include limb weakness with prominent bulbar weakness and tongue amyotrophy;^7,9^ isolated motor neuropathy with no clinical or neurophysiological evidence of sensory involvement;^8,10,15,16^ or a sensorimotor polyneuropathy with axonal^2,3,17-19^ or mixed axonal and demyelinating features^1,20^ similar to that which was observed in our patient (Table 2). While upper and lower motor neuron involvement can bring these adolescents or young adults to the attention of neurologists,^14^ it is the nonneurological features including adrenal insufficiency that has the potential to give rise to life-threatening complications. Our patient did report fatigue and show signs of skin pigmentation which allowed for the prompt identification of adrenal insufficiency. Hypoadrenalism may be life-threatening if an inadequate endogenous adrenal response occurs with a physiological stressor such as an intercurrent illness, trauma, or surgical intervention. Although we initially considered a peroxisomal disorder in this boy, exome sequencing enabled the diagnosis of Allgrove syndrome to be made.

**Table 2.** Neurophysiological (NCS/EMG) Findings in Allgrove (Triple A) Syndrome Patients.

Ref:	N	Age(s)	Sensory NCS	Motor NCS	Interpretation:
Goizet^7^	1	33 years	Normal	Normal^a^	Motor neuron disease
Miyazawa^9^	1	38 years	Normal	Low CMAP amplitudes to ulnar and tibial nerves	Motor neuron disease
Strauss^8^	1	22 years	Normal	Low CMAP amplitudes to median, ulnar, tibial nerves; CV = normal.	Motor neuropathy with axonal features
Messina^15^	1	10 years	Normal	Low CMAP amplitudes to median, ulnar, peroneal. Peroneal CV = 32 m/s (80% LLN)	Motor neuropathy with axonal features
Dixit^16^	1	11 years	Normal	Low CMAP amplitude to median, peroneal.	Motor neuropathy with axonal features
Koehler^10^	1	14 years	Normal	Normal at 3 yo; developed low CMAP amplitude to median, ulnar & peroneal nerves by age 9 yo. CV = normal	Motor neuropathy with axonal features
Houlden^2^	6	18-48 years	Absent/low SNAP amplitude to median (3/6); ulnar (4/6); sural (3/6)^b^	Low CMAP amplitude to median (3/6); peroneal (5/6)^b^	Sensorimotor polyneuropathy (motor predominant) with axonal features
Kimber^17^	3	40-60 years	Absent/low SNAP amplitude to median (3/3); ulnar (3/3); sural (3/3)	Absent/low CMAP amplitude to median (1/3); ulnar (1/3); peroneal (1/3); tibial (1/2); CV = >80% LLN	Sensorimotor polyneuropathy with axonal features
Vishnu^18^	1	36 years	Low SNAP amplitude to median, ulnar, radial SNAP amplitude; Absent sural response.	Low CMAP amplitudes to median, peroneal CV = >74% LLN.	Sensorimotor polyneuropathy with axonal features
Vallet^3^	8	21-59 years	Low SNAP amplitudes to median (6/8 patients) and ulnar (5/7 patients)	Low CMAP amplitudes to median (3/8); ulnar (8/8); tibial (8/8). CV = within range of axonal loss	Sensorimotor polyneuropathy with axonal features
Gebril^19^	2	13, 15 years	Mixed sensory and motor axonal neuropathy with decreased amplitudes		Sensorimotor polyneuropathy with axonal features
Nakamura^20^	1	60 years	Low median SNAP amplitude; absent sural response	Low median & tibial CMAP amplitudes. CV = 62 = 67% LLN.	Sensorimotor polyneuropathy with axonal and demyelinating features
Dumic^1^	2	3-5 years	Mixed sensory and motor demyelinating neuropathy. CV = 67-76% LLN		Sensorimotor polyneuropathy with demyelinating features
Current	1	11 years	Absent sural, peroneal response; low median, ulnar SNAP amplitude;	Low CMAP amplitude to ulnar, peronal, tibial. CV 52 to 86% LLN	Sensorimotor polyneuropathy with axonal and demyelinating features

Abbreviations: EMG, electromyography; SNAP, sensory nerve action potential; CMAP, compound motor; action potential; CV, conduction velocity; LLN, lower limit of normal; yo, years old.

^a^ EMG noted diffuse denervation.

^b^ Progressive decline in amplitude with serial studies performed.

Patients with isolated motor nerve or motor neuron involvement, particularly those with prominent bulbar involvement have the potential to be mistaken for amyotrophic lateral sclerosis or other motor neuronopathies given the frequent association of upper and lower motor neuron symptoms that are present.^7,8^ Serial studies of individual patients have documented a progressive decline in CMAP amplitude over time.^2,9^ One report documented a serial decline in the sural sensory nerve action potential (SNAP) amplitude from 10 to 16 years old,^2^ while in another the sensory responses remain normal and unchanged from 9 to 14 years of age.^9^

Nerve biopsies have been reported in at least 4 affected patients. A 10-year-old boy and a 35-year-old man demonstrated a normal sural nerve biopsy despite a muscle biopsy showing prominent neurogenic changes.^21,22^ Each patient showed a normal sensory examination and isolated motor nerve findings on neurophysiological testing. A separate report of a 40-year-old man who had symptom onset at 5 years of age noted his sural nerve biopsy to show changes consistent with axonal degeneration with marked loss of myelinated axons and some unmyelinated axons.^17^ The sural nerve of a 45-year-old woman who developed achalasia in her 20s revealed a severe axonal neuropathy with secondary demyelination.^22^ These biopsy findings suggest that motor and axonal findings may predominate in many patients and that the demyelinating changes, when present, may potentially be a secondary phenomenon.

The neurophysiological findings in Allgrove syndrome are heterogeneous ranging from a motor neuronopathy or motor-predominant neuropathy to a sensorimotor polyneuropathy with axonal and demyelinating features. Given this heterogeneity, it is particularly important that nonneurological symptoms such as alacrima, achalasia, and Addison disease be considered since they may provide important clues to facilitate a timelier diagnosis and avoid potential life-threatening complications that can occur with this rare disease. Like many cases of Allgrove syndrome reported in the literature, our patient did show alacrima as well as skin pigmentation which can be a sign of adrenal insufficiency. Other symptoms of adrenal insufficiency can include chronic fatigue and weakness which have the potential to be misattributed to underlying neuromuscular findings. Obtaining a morning cortisol level or an ACTH stimulation test should be considered to assist with making a definitive diagnosis but also to avoid a life-threatening adrenal crisis that can occur.

## Author Contributions

DIW drafted the manuscript, contributed to the conception and design of the case report, and gave final approval. HJM contributed to the writing of the manuscript, the conception and design of the case report, and agrees to be accountable for all aspects of work ensuring integrity and accuracy. MKG, KDK, MO, YI, TH, KMB, ML were responsible for exome sequencing, analysis, and interpretation. HJM, MKG, KMB, KDK, ML critically revised the manuscript, gave final approval, and agree to be accountable for all aspects of work ensuring integrity and accuracy.
